# In Vitro Studies of Nanoparticles as a Potentially New Antimicrobial Agent for the Prevention and Treatment of Lameness and Digital Dermatitis in Cattle

**DOI:** 10.3390/ijms24076146

**Published:** 2023-03-24

**Authors:** Magdalena Kot, Aleksandra Kalińska, Sławomir Jaworski, Mateusz Wierzbicki, Sebastian Smulski, Marcin Gołębiewski

**Affiliations:** 1 Animal Breeding Department, Warsaw University of Life Sciences, 02-786 Warszawa, Poland; 2 Department of Nanobiotechnology, Warsaw University of Life Sciences, 02-786 Warszawa, Poland; 3 Department of Internal Diseases and Diagnostics, Poznań University of Life Sciences, 60-637 Poznań, Poland

**Keywords:** silver, copper, gold, platinum, iron, nanoparticles, nanocomposites, lameness, digital dermatitis, dairy cows

## Abstract

Digital dermatitis (DD) is the second most prevalent disease in dairy cattle. It causes significant losses for dairy breeders and negatively impacts cows’ welfare and milk yield. Despite this, its etiology has not been entirely identified, and available data are limited. Antibiotic therapy is a practical method for managing animal health, but overuse has caused the evolution of antibiotic-resistant bacteria, leading to a loss in antimicrobial efficacy. The antimicrobial properties of metal nanoparticles (NPs) may be a potential alternative to antibiotics. The aim of this study was to determine the biocidal properties of AgNPs, CuNPs, AuNPs, PtNPs, FeNPs, and their nanocomposites against pathogens isolated from cows suffering from hoof diseases, especially DD. The isolated pathogens included *Sphingomonas paucimobilis*, *Ochrobactrum intermedium* I, *Ochrobactrum intermedium* II, *Ochrobactrum gallinifaecis*, and *Actinomyces odontolyticus*. Cultures were prepared in aerobic and anaerobic environments. The viability of the pathogens was then determined after applying nanoparticles at various concentrations. The in vitro experiment showed that AgNPs and CuNPs, and their complexes, had the highest biocidal effect on pathogens. The NPs’ biocidal properties and their synergistic effects were confirmed, which may forecast their use in the future treatment and the prevention of lameness in cows, especially DD.

## 1. Introduction

Modern livestock production is significantly different to the realities of farming over the last 15 to 20 years, even though this is a relatively short period of time. The continuously growing demand for animal products has made it necessary to boost production to meet global consumer needs. Activities aimed at maximizing livestock performance have been initiated to this end [1]. The incidences of lameness reflect the misconduct of dairy producers and a failure of duty associated with proper breeding practices, which has led to poor hoof health. Unfortunately, these actions, including negligence, have resulted in cows suffering pain associated with lameness, which has led to a deterioration in animal health status and welfare [2]. Lameness is one of the most common diseases found in dairy herds, and it results, globally, in the greatest financial losses to breeders [3]. It is mainly caused by bacterial infections, which lead to hoof dermatitis and clinical lameness [4].

Digital dermatitis (DD) is a polybacterial infectious foot disease complex caused by multiple bacterial agents [5]. Important etiological factors for lameness include *Treponema* spp. such as *T. denticola*, *T. maltophilum*, *T. medium*, *T. putidum*, *T. phagedenis*, and *T. paraluiscuniculi. Treponema* spp. are the most frequently isolated etiological agent for DD; however, the available literature also indicates other isolates, such as *Guggenheimella* spp., *Borrelia* spp., *Porphyromonas* spp. [6], *Bacteroides* spp., *Campylobacter* spp., *Fusobacterium necrophorum*, and *Dichelobacter nodosus* [7].

Despite the prevalence of digital dermatitis, the etiological agent and microbial composition are not completely determined, and the results from the available literature are not consistent [8]; therefore, in order to explore the bacterial factors that result in DD, it is necessary to conduct further research to expand our knowledge of it.

Various approaches are used to treat DD, but the most common forms, due to the location of the infection, are hoof baths and bandage dressings. In both cases, however, antibiotics are used [5]. It is more common to use localized antibiotic therapy than a general treatment, but reports vary, indicating that antibiotic treatment is not sufficiently effective in all cases. Tetracycline, oxytetracycline, erythromycin, lincomycin, and tiamulin are the most common antibiotics used in footbaths [9]; non-antibiotic alternatives include copper sulfate, formalin, peracetic acid, and organic acid solutions. Unfortunately, despite their antibacterial properties, some of these treatments adversely affect the animals by irritating the skin [10].

Previous studies have indicated that antibiotic use in the treatment of digital dermatitis does not provide consistently successful results [11]. Consequently, an effective treatment for digital dermatitis cannot be clearly determined due to the limited effect of the antibiotics and the complexity of the polybacterial infectious disease. As a result, it is crucial that we look for new, alternative solutions that will help effectively treat bacterial infections. One of these solutions is the use of nanotechnology, especially metal nanoparticles.

Both the physical and chemical properties of nanoparticles differ depending on the particle, which affects their antimicrobial properties. The differences in antibacterial activity are affected by the nanoparticles’ surface area to volume ratio [12], shape, size (1–100 nm) [13], and surface properties such as the zeta potential [14].

Previous reports have confirmed nanoparticles’ antibacterial activity against medically important pathogens, including those species that are resistant to conventional treatments—this demonstrates their potency and efficacy. Silver nanoparticles (AgNPs) are effective, even against multidrug-resistant (MDR) bacteria such as methicillin-resistant *Staphylococcus aureus* [15] and *Staphylococcus epidermidis* [16], erythromycin-resistant *Streptococcus pyogenes*, ampicillin-resistant *Escherichia coli*, and multidrug-resistant *Pseudomonas aeruginosa* [17]. AgNPs also reduce biofilm formation in *S. aureus* [18]. Moreover, gold nanoparticles (AuNPs) destroy the MDR cells of *P. aeruginosa* [19]. These strong antimicrobial properties suggest that nanoparticles could be used as an alternative to antibiotics, and may become an innovative agent against various pathogens, among other bacteria isolated from diseased hooves. The available references indicate that there are variations associated with the biocidal properties of nano-platinum (PtNP) based on their method of synthesis [20]; for instance, green synthesized PtNPs effectively destroy bacterial cells [21]. The literature also confirms the antibacterial effects of green synthesized iron (FeNPs) [22] and copper (CuNPs) nanoparticles [23]. 

Nanoparticle complexes demonstrate synergistic effects towards each other, enhancing their antimicrobial properties; however, these effects have not been entirely established for all combinations due to the variety of nanoparticles. The most important fact is that nanoparticles have no toxic influence on bovine or human cells [24], which is crucial for animal and human safety if we are going to consider using nanoparticles in the future as a potential disinfectant product.

The main goal of the current research was to determine the biocidal effects of AgNPs, CuNPs, AuNPs, PtNPs, FeNPs, and their nanocomposites on pathogens isolated from tissue samples, pus, pus and blood, and other swabs from the limbs of cattle diagnosed with lameness. The isolated pathogens included *Sphingomonas paucimobilis*, *Ochrobactrum intermedium* I and *Ochrobactrum intermedium* II, *Ochrobactrum gallinifaecis*, and *Actinomyces odontolyticus*. These microbes have been associated with DD for the first time, which is significant, and contributes greatly to the literature and to scientific development. The conducted experiment also determined the main characteristics of both the selected nanoparticles and their complexes, such as morphology, zeta potential, size, and hydrodynamic size distribution.

Despite DD being among the most frequent diseases in cattle herds, the etiological agents and the alternatives for its treatment have not been sufficiently established. The described correlations suggest that the use of multiple types of NPs could be a beneficial solution for the control of the microorganisms responsible for DD, while the results of the study will improve our knowledge of DD and present a possible alternative for its treatment and prevention.

## 2. Results

### 2.1. Identification of the Isolated Microorganisms

In the present study, the Gram-negative aerobic bacteria Sphingomonas paucimobilis, Ochrobactrum intermedium I and Ochrobactrum intermedium II, and Ochrobactrum gallinifaecis, and the Gram-positive anaerobic Actinomyces odontolyticus were isolated from biological material obtained from the limbs of cattle diagnosed with lameness. Two analytical instruments, the MALDI-TOF MS and the VITEK 2, were used to identify the isolated microorganisms; this made the identification precise and reliable. Nowadays, the MALDI-TOF MS is used in clinical microbiology laboratories because of its potential for the development of matrix-assisted laser desorption ionization time-of-flight mass spectrometry. The VITEK 2 is a compact system that uses colorimetric reagent cards that are incubated and interpreted automatically using a database containing well-described strains. This fact is important, especially as the isolated pathogens had not previously been connected with the digital dermatitis disease. The obtained results were, therefore, more precise than standard microbiology analysis. Table 1 and Table 2 present the extracted report information that enabled the identification of the isolated microorganisms.

### 2.2. Determination of the Nanoparticles’ Morphology and Their Complexes

**Figure 1:**
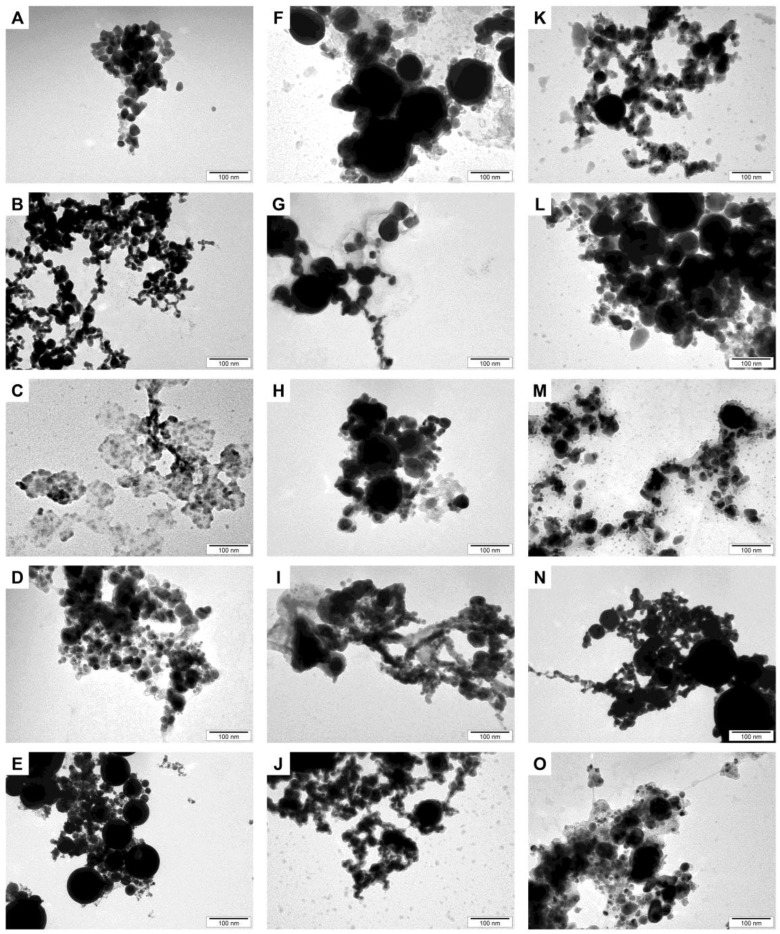
Transmission electron microscopy (TEM) images of the studied nanoparticles and their complexes. (**A**)—AgNPs, (**B**)—AuNPs, (**C**)—CuNPs, (**D**)—FeNPs, (**E**)—PtNPs, (**F**)—AgFeNPs, (**G**)—AuAgNPs, (**H**)—AuFeNPs, (**I**)—CuAgNPs, (**J**)—CuAuNPs, (**K**)—CuPtNPs, (**L**)—CuFeNPs, (**M**)—PtAgNPs, (**N**)—PtAuNPs, (**O**)—PtFeNPs.

All nanoparticles, apart from CuNPs, presented in Figure 1, have a spherical morphology. In contrast, it was difficult to estimate the morphology of CuNPs due to contaminations and their size. The CuNPs and their complexes contained contaminants, probably in the form of copper salts formed by the dissolution of the nanoparticles.

### 2.3. Determination of Physicochemical Properties of Nanoparticles and Their Complexes

The data presented in Table 3 show that the zeta potential for most of the NPs achieved negative values (from −3.66 to −26.2 mV), while CuNPs and CuAgNPs achieved positive values (7.75 mV and 15.63 mV, respectively). PtNPs achieved values close to 0 (−3.66 mV), while the value that was found to be farthest from 0 was for AgNPs (−26.2 mV). 

The hydrodynamic diameter represents the average agglomerate size, which varies depending on the type of nanoparticle. Certain nanoparticles tended to form larger agglomerates, including single nanoparticles such as CuNPs, and FeNPs, but also complexes like AuAgNPs and AgFeNPs. The nanoparticle with the least tendency to agglomerate was PtNPs.

The sizes of the studied NPs differed from each other. The largest sizes were characteristic for FeNPs and PtNPs (5–100 nm), while AgNPs and AuNPs were smaller (10–50 and 10–40 nm, respectively). The smallest sizes were typical for CuNPs (1–10 nm). The obtained results are presented in Table 4.

### 2.4. Biocidal Properties of AuNPs, AgNPs, PtNPs, CuNPs, and FeNPs against Microorganisms Isolated from Hoof Lesions

Bacterial cell viability was presented as a percentage [%], where the control group was always stated as 100%. The bacterial cell viability of the control group was compared to the bacterial cell viability of the experimental groups, and the obtained results are presented in Table 5.

These studies indicated that there were differences in the viability of pathogens after the nanoparticles were applied, depending on the pathogen and nanoparticle concentrations. Ag, Au, and Cu nanoparticles demonstrated the strongest biocidal activity against the tested microorganisms when they were in higher concentrations (12.5–25 ppm). The highest concentration of AuNPs (25 ppm) reduced the viability of three strains, *Sphingomonas paucimobilis*, *Ochrobactrum gallinifaecis*, and *Actinomyces odontolyticus*, by 14.80%, 14.76%, and 6.92%, respectively. In the case of AgNPs and CuNPs, a strong biocidal effect was observed at the lower concentrations of 12.5 ppm and 6.25 ppm, respectively. AuNPs, at concentrations of 1.56 ppm and 3.125 ppm, promoted increases in selected pathogens by an insignificant amount, excluding *Ochrobactrum intermedium* I; concentrations of CuNPs at 1.56 ppm promoted the growth of *Actinomyces odontolyticus*. *Sphingomonas paucimobilis* was the most susceptible pathogen to the antibacterial effects of the selected NPs. AgNPs exhibited the most effective antibacterial activity, while, in contrast, platinum and iron nanoparticles had the least activity—in some instances even promoting bacterial growth. FeNPs and PtNPs, therefore, were not used in the latter part of the experiment.

### 2.5. Biocidal Properties of AuNP, AgNP, and CuNP Complexes against Microorganisms Isolated from Hoof Lesions

Data regarding the biocidal properties of NPs complexes against isolated bacteria are presented in Table 6. There was a decrease in the levels of pathogens for all nanoparticle complexes. Lower concentrations of nanocomplexes showed more potent biocidal properties in comparison to the results from individual nanoparticles. Nevertheless, higher concentrations (12.5 ppm) of nanoparticle complexes showed more powerful antibacterial activity than lower concentrations (0.78 ppm). The complex that demonstrated the strongest antibacterial effect was AgCuNPs, whereas the CuAuNP complex had the weakest effect. The AgCuNP complex, at a concentration of 6.25 ppm, decreased the pathogens’ viability to 1.5–5%. Therefore, further studies should probably use this at higher concentrations, especially in herd conditions. As with the effects of individual nanoparticles, *Sphingomonas paucimobilis* was most susceptible to the complexes, while *Ochrobactrum intermedium* II was the most resistant. Nevertheless, the levels of all pathogen viabilities were decreased using the selected nanoparticle complexes.

## 3. Discussion

In recent years, antibiotic resistance has become a significant problem, affecting both animals and humans [25,26]. Nowadays, researchers and breeders are facing new challenges, and one of these is the growing number of pathogens that are resistant to the most popular drugs. The overuse of antibiotics has led to this resistance [27]. These drugs were used not only in the treatment of farm animals, but also often as a prevention method [28,29]. Antibiotics that were widely abused have now lost their efficacy, and there is a lack of new, effective antibiotics to replace them [30]. Due to their properties, nanoparticles have a broad range of uses and high potential in diverse fields; however, their properties are dependent on their physicochemical characteristics. Previous studies have also confirmed their antiviral, antifungal, and antiparasitic properties [31]. 

Most significantly, nanoparticles have a strong affinity for bacterial cells, resulting in protein denaturation, and contribute to the formation of free radicals that lead to the disruption of DNA replication and pathogen protein expression [32]. They also damage cell membranes and mitochondria, leading to cell death [33]. Crucially, the bacterial cell is incapable of developing resistance to nanoparticles. Therefore, as an antimicrobial agent, they can be a great alternative to antibiotics [34]. 

In a study by Berry et al. [11], lincomycin HCl soluble powder was used in the treatment of digital dermatitis in the form of a paste under a dressing; however, after 11 months of antibiotic therapy, the disease recurred. In addition, the treatment of different forms of digital dermatitis has resulted in different outcomes. The use of oxytetracycline for infection treatment of the interdigital cleft was less effective compared to using it to treat lesions on the heels [35]; therefore, it is challenging to recommend an effective treatment for DD.

Results from Wernicki et al. suggested that the antibacterial properties of PtNPs depended on the method used to synthesize them. There are reports by Wernicki et al. [20] that suggest that antimicrobial properties were lacking in PtNPs synthesized using physical methods. In this study, we used PtNPs obtained by physical methods, and their effect on isolated lameness bacteria confirmed the findings reported by Wernicki et al., indicating that their antibacterial properties were quite low. Moreover, in the case of bacteria associated with lameness, not only was PtNPs’ poor antibacterial effect observed, but it also promoted the bacteria’s growth. However, it is worth noting that the study by Wernicki et al. was conducted on mastitic bacteria isolated from cattle. Moreover, there is a shortage of cases to refer to in the available literature. The results of our study concerning the effect of PtNPs were similar to FeNPs, resulting in an increase in bacterial viability. The available literature indicates that green synthesized nanoparticles have antibacterial properties [22], while those we used in our research promoted the growth of bacteria (except for *O. intermedium* I, where the viability slightly decreased).

The biocidal efficacy of nanoparticles depends on, among other factors, the cell wall structures of the bacteria, which is related to whether the bacteria is Gram-negative (G-) or Gram-positive (G+) [36]. The literature indicates that AuNPs show antibacterial activity against both G- and G+ bacteria, however, this is dependent on the AuNPs concentration. According to Shamaila et al. [37], lower concentrations were sufficient to demonstrate the antibacterial activity of AuNPs against G- bacteria but not against G+ bacteria. The current study did not confirm these results, in which the most resistant bacteria to AuNPs were the G- *O. intermedium* II, *S. paucimobilis*, and *O. gallinifaecis* and the G+ bacterium *A. odontolyticus*. Low concentrations of nanocolloids promoted the growth of bacteria, except for *O. intermedium* I, where even the lowest concentration (1.56 ppm) showed some minimal antibacterial activity. There was a decrease in pathogen titers for all bacterial isolates for concentrations of 12.5–25 ppm. A concentration of 25 ppm demonstrated the strongest biocidal activity against G+ *A. odontolyticus* bacteria.

The available publications indicate that AgNPs exhibit antibacterial activity against both G- and G+ bacteria [38]. The results obtained in this experiment confirmed these results. After the application of AgNPs, a reduction in the quantities of each pathogen was observed to varying degrees, according to the concentration of AgNPs. The G+ *A. odontolyticus* was the most resistant bacteria to the activity of these nanoparticles, whereas G- bacteria were the most susceptible.

Alizadeh et al. [39] investigated the effects of AgNPs on G- bacteria. The study demonstrated that they were more effective at high concentrations (>10 ppm) compared to low concentrations (<10 ppm). Han et al. [40] found that AgNPs at concentrations of 0.06–0.98 ppm showed antibacterial activity against both G- and G+ bacteria. In the current study, the efficacy of AgNPs was correlated with their concentration, and they were effective even at the lowest concentration (1.56 ppm), confirming the results from Alizadeh et al. and Han et al.

Regarding the effectiveness of CuNPs, there are certain discrepancies in the literature associated with the relationship between the way they are synthesized and their biocidal capacity. Shankar et al. [41] applied green-synthesized CuNPs, which showed antibacterial activity against both G- and G+ bacteria; Chowdhury et al. [42] used nanoparticles obtained using the chemical reduction method. Their nanoparticles showed stronger antibacterial activity against G- than G+ bacteria, which is consistent with our results. The G+ bacterium *A. odontolyticus* exhibited the strongest resistance to CuNPs, in contrast to G- bacteria. A study conducted by Dugal et al. [43] indicated CuNPs’ antibacterial potential against G- bacteria at concentrations of 19.60 ppm, which is also consistent with the results of the current study. Despite the high agglomerate-forming potential of the CuNPs (580.7 nm) used in our experiment, they exhibited strong antibacterial properties, which may be related to their small size (1–10 nm).

A study by Kalińska et al. [24] showed AgNPs and CuNPs’ antibacterial properties against G+ and G- bacteria. Their AgNPs had a similar zeta potential value to the results of the conducted experiment. However, there were noticeable differences in the zeta potential of CuNPs, which had values from −20.93 to −9.25 mV, while in the current experiment the value was 7.75 mV. There were also differences in the sizes of the NPs. AgNPs in this experiment measured 10–50 nm, while those used by Kalińska et al. were almost twice this size (35–100 nm). In the case of CuNPs, which in our experiment were 1–10 nm, those used by the by Kalińska et al. were 0.5–80 nm. Differences also appeared in the hydrodynamic diameters of NPs, whereas in our study the AgNPs used in the experiment showed less potential for forming agglomerates. At 154.5 nm, the potential was 262 nm for Kalińska et al. The CuNPs in our study showed almost double the potential (580.70 nm) in comparison with the results from Kalińska et al. (288.6 nm). Nevertheless, both the nanoparticles in our study and Kalińska et al.’s studies showed good bactericidal activity. Despite these differences, AgNPs and CuNPs both showed antibacterial activity, which is consistent with the results of our study. Lange et al. [44] used AgNPs that had very similar zeta potential (−26.7 mV) and hydrodynamic diameter (154.1 nm) values to the nanoparticles used in our experiment. However, the zeta potential of the AgCuNP complex (−9.09 mV) in the studies by Lange et al. differed significantly from the value for the complex obtained from our experiment, which took a positive value of 15.63 mV. Despite this, the antimicrobial activity against G+ and G- bacteria in Lange et al.’s study and our study was similar. Research by Belyakova et al. [45] indicated that CuNPs and AgNPs had antibacterial activity against *Fusobacterium necrophorum*, *Treponema* spp., and *Borrelia* spp. bacteria, which are etiological factors that lead to lameness. Our results confirmed these nanoparticles’ strong antibacterial activity against the isolated bacteria associated with lameness.

AuNPs were found to promote growth in the studied bacteria, which was not confirmed by other sources in the available literature, including bacteria not even associated with lameness. However, there are available reports that indicate that AuNPs had no antibacterial activity, or that they exhibited biocidal activity only at high concentrations, which is consistent with our results [46]. There are also reports that AuNPs exhibited antimicrobial activity against G- and G+ bacteria, but this was due to the matching of the nanoparticles’ functional groups [19], which was not applied in our experiment. In contrast, the AuAgNP and AuCuNP complexes showed strong antibacterial properties even at low concentrations. Similar synergistic interaction results were observed by Lange et al. [44], who pointed out that nanoparticles can disrupt bacteria biofilm and that this phenomenon is important in, for example, bovine mammary gland inflammation.

The available literature is very limited concerning the bacteria associated with DD, and the bacteria isolated for this study have never been associated with digital dermatitis. Therefore, the authors compared the effect of metal NPs on microorganisms, differentiating them in relation to the structural differences between the walls of gram-negative and gram-positive bacteria, which affected their susceptibility to external environmental factors. Isolating and making cultures of these pathogens is challenging, which means the number of available sources is limited and the issue itself is under-researched. Therefore, it is exceedingly difficult to address the reports of other authors. On the other hand, this fact also suggests the importance of conducting further research in this field.

Perhaps the discrepancies in the effects of nanoparticles on the aerobic and anaerobic species that were isolated from diseased hooves were due to the anaerobic species’ nature, the structure of the cells and their composition, the metabolic processes of the bacteria, and the characteristic features of the species [33]. In any case, there has been no research in this direction, and this requires further development of the topic. 

## 4. Materials and Methods

### 4.1. Isolated Bacteria Cultures

Biological material (tissue samples, pus, pus and blood, and other swabs from the cattle diagnosed with lameness) was placed in a saline solution and then inserted into a Stuart orbital incubator SI500 shaker (Reagecon Diagnostics Ltd., Shannon, Ireland) for 24 h at 5 °C. The resulting suspension was seeded onto a Columbia agar + blood/Chocolate agar, CASA (Argenta, Poznań, Poland) and onto brain–heart agar (Biomaxima, Lublin, Poland). The cultures were placed in aerobic and anaerobic environments to maximize the possibility of increasing the numbers of the different isolated pathogen species. The bacterial culture was processed in an aerostat using a GENbag anaer atmosphere generator (bioMérieux, Warsaw, Poland). Material from the cultivated colonies was diagnosed using a MALDI-TOF MS (Bruker, Poznań, Poland) and a VITEK 2 (bioMérieux, Warsaw, Poland) to verify the species that had been isolated.

### 4.2. Nanoparticles

Commercially available nanoparticles, AuNPs, AgNPs, CuNPs, FeNps, and PtNPs, which were used in the experiment, were obtained from Nano-tech (Warsaw, Poland). Nanoparticle hydrocolloids were sonicated for 2 min at 500 W and 20 kHz before their use in tests.

### 4.3. Determination of the Nanoparticles’ Morphology

The morphology of the nanoparticles obtained from Nano-Tech Poland was determined by analyzing transmission electron microscopy (TEM) images. The nanoparticles were viewed using a JEM-2000EX transmission electron microscope (JEOL). The nanoparticles’ hydrocolloid was sonicated for two minutes and then applied to a molding TEM grids (Formvar on 3 mm 200 mesh Cu grids, Agar Scientific, Stansted, UK) and left to dry for 24 h. Images of the nanoparticles were taken at a voltage of 80 keV.

### 4.4. Determination of the Physicochemical Properties of Nanoparticles

The zeta potential and size distribution were determined using a Zetasizer Nano ZS (ZEN3500, Malvern Instruments, Malvern, UK). The average size of selected nanoparticles was measured by transmission electron microscopy and by dynamic light scattering (DLS) in order to determine the hydrodynamic size (average agglomerate size).

### 4.5. Determination of Bacterial Viability after Treatment with Nanoparticles and Their Complexes

A suspension of bacteria was prepared from each isolate at an optical density of 0.5 on the McFarland scale (1.5 × 10^8^ CFU/mL). The suspension was pipetted into a 96-well plate at a volume of 50 µL per well. The experiment used commercially available AuNPs, AgNPs, CuNPs, FeNPs, and PtNPs (Nano-tech, Warsaw, Poland), which were extracted using physical methods. Following that, 50 µL each of nanoparticle hydrocolloids were added so that the final NP concentrations were as follows: for single nanoparticles—1.56, 3.125, 6.25, 12.5, and 25 ppm; for PtNPs—0.625, 1.25, 2.5, 5, and 10 ppm. The concentrations for the nanocomplexes were 0.78, 1.56, 3.125, 6.25, and 12.5 ppm. Incubation was carried out for 24 h at 37 °C under anaerobic conditions. After 24 h, 10 µL of Presto Blue reagent (Invitrogen, Waltham, MA, USA) was added to each well and the absorbance was read spectrophotometrically at 570 nm (Tecan, Durham, NC, USA). Viability was determined from the following formula:

$$\frac{\mathrm{absorbance}\;\mathrm{of}\;\mathrm{the}\;\mathrm{experimental}\;\mathrm{sample}}{\mathrm{absorbance}\;\mathrm{of}\;\mathrm{the}\;\mathrm{control}\;\mathrm{sample}}\times 100\%$$

The data were analyzed using one-way analysis of variance (ANOVA) in STATGRAPHICS^®^ Centurion XVII, version 17.2.05 software (StatPoint Technologies, Inc., Warrenton, VA, USA).

## 5. Conclusions

The conducted in vitro study confirmed that AuNPs, AgNPs, and CuNPs have antibacterial activity against selected digital dermatitis pathogens and confirms their synergistic effect, however their effects varied according to their concentrations. AgNPs and CuNPs exhibited the strongest antibacterial properties, whereas the most active complex was AgCuNPs (*p* ≤ 0.05). The highest concentration tested for AuNPs was 25 ppm, and this reduced the viability of *Sphingomonas paucimobilis*, *Ochrobactrum gallinifaecis*, and *Actinomyces odontolyticus* by 14.80%, 14.76%, and 6.92%, respectively. In the case of AgNPs and CuNPs, a strong biocidal effect was observed at the lower concentrations of 12.5 ppm and 6.25 ppm, respectively. The AgCuNP complex, at concentrations of 6.25 and 12.5 ppm, decreased pathogen viability to 1–6%. PtNPs and FeNPs showed very weak antibacterial activity, and, in certain cases, selected NPs that promoted bacterial growth; in contrast, all nanocomplexes reduced pathogen viability. The study suggests that AgCuNPs may provide an alternative to antibiotics in the treatment of digital dermatitis, but further in vivo experiments are required to confirm their biocidal properties under herd conditions. 

## Figures and Tables

**Table 1:** Pathogens identified using the MALDI-TOF MS, including score value and NCBI identifier.

Matched Pattern	Score Value	NCBI Identifier
*Ochrobactrum gallinifaecis*	1.76	215590
*Ochrobactrum intermedium* I	2.0	94625
*Ochrobactrum intermedium* II	1.78	94625

**Table 2:** Pathogens identified using the VITEK 2, including the biochemical information for the identification of the isolated microorganisms.

**Identified Microorganism**	*Actinomyces odontolyticus*											
**Biochemical properties**	dGAL	-	LeuA	+	ELLM	+	PheA	+	dMNE	-	BGURi	-
	dCEL	+	TyrA	+	APPA	-	dGLU	-	URE	-	PVATE	-
	SAC	-	ARB	-	NAG	-	BGLUi	-	ARG	-	AIFUC	-
	BGALi	-	AARA	-	AGALi	-	BMAN	-	AMANi	-	dXYL	+
	MTE	-	ESC	+	BdFUC	-	BNAGi	-	AARAF	+		
	PHOS	+	IARA	-	dRIB2	-	OPS	-	PyrA	-		
	GRAM	+	MORPH	-	AERO	+	ProA	+	dMAL	+		
**Identified microorganism**	*Sphingomonas paucimobilis*											
**Biochemical properties**	APPA	-	ADO	-	PyrA	-	IARL	-	dCEL	+	BGAL	-
	H2S	-	BNAG	-	AGLTp	-	dGLU	+	GGT	-	OFF	-
	BGLU	-	dMAL	+	dMAN	-	dMNE	+	BXYL	-	BAIap	-
	ProA	-	LIP	-	PLE	-	TyrA	+	URE	+	dSOR	-
	SAC	-	dTAG	-	dTRE	+	CIT	-	MNT	-	5KG	-
	ILATk	-	AGLU	+	SUCT	-	NAGA	-	AGAL	-	PHOS	-
	GlyA	-	ODC	-	LDC	-	IHISa	-	CMT	-	BGUR	-
	O129R	-	GGAA	-	IMLTa	-	ELLM	+	ILATa	-		

“+” found concordance of biochemical characteristics of the bacteria; “-“ not found concordance of biochemical characteristics of the bacteria.

**Table 3:** Physicochemical properties of selected nanoparticles and their complexes.

Nanoparticle	Zeta Potential (mV)		Hydrodynamic Diameter (nm)	
	Values	Average	Values	Average
Ag	−26.30	−26.20	157.20	154.50
	−25.80		152.10	
	−26.50		154.20	
Cu	4.54	7.75	987.00	580.70
	8.80		363.00	
	9.92		392.10	
Pt	−3.10	−3.66	221.10	171.23
	−3.09		148.80	
	−4.79		143.80	
Au	−17.50	−18.00	201.50	189.50
	−18.40		187.90	
	−18.10		179.10	
Fe	−19.10	−18.83	300.70	311.27
	−19.10		314.70	
	−18.30		318.40	
AuAg	−21.50	−20.57	518.00	455.67
	−18.90		425.60	
	−21.30		423.40	
PtAg	−24.50	−23.80	179.60	178.70
	−21.40		183.70	
	−25.50		172.80	
FeAg	−16.50	−15.87	366.40	353.53
	−15.90		350.40	
	−15.20		343.80	
CuAg	14.70	15.63	238.20	243.27
	16.90		244.50	
	15.30		247.10	

**Table 4:** Size distribution of nanoparticles based on TEM images.

Nanoparticle	Size (nm)
AgNPs	10–50
CuNPs	1–10
FeNPs	5–100
PtNPs	5–100
AuNPs	10–40

**Table 5:** Viability analysis of the microorganisms isolated from ulcerated hoof sores and exposed to Au, Ag, Pt, Cu, and Fe nanoparticles.

Nanoparticles (ppm)	*Sphingomonas paucimobilis*	*Ochrobactrum intermedium* I	*Ochrobactrum intermedium* II	*Ochrobactrum gallinifaecis*	*Actinomyces odontolyticus*
**Control group**	100 ± 3.6	100 ± 4.2	100 ± 2.25	100 ± 7.86	100 ± 0.01
Au 1.56	102.12 ± 3.54	86.17 ± 4.3	101.86 ± 4.2	106.96 ± 12.22	103.57 ± 1.44
Au 3.125	104.4 ± 2.9	85.64 ± 7.9	103.25 ± 3.8	101.9 ± 15.06	104.6 ± 1.23
Au 6.25	95.4 ± 2.6	76.76 ± 3.1 *	101.72 ± 1.9	101.64 ± 3.98	104.92 ± 0.27
Au 12.5	94.2 ± 3.7	62.15 ± 4.6 *	99.8 ± 3.6	70.68 ± 7.29 *	68.68 ± 7.69 *
Au 25	14.8 ± 0.6 *	42.4 ± 1.2 *	95.2 ± 3.7	14.76 ± 0.95 *	6.92 ± 0.9 *
Ag 1.56	7.12 ± 0.08 *	16.8 ± 1.8 *	8.21 ± 0.3 *	12.82 ± 0.33 *	47.96 ± 8.83 *
Ag 3.125	4.11 ± 0.51 *	7.6 ± 0.2 *	6.63 ± 0.8 *	12.24 ± 0.29 *	36.56 ± 2 *
Ag 6.25	3.37 ± 0.08 *	5.4 ± 0.3 *	5.52 ± 0.6 *	13.39 ± 0.94 *	28.73 ± 5.31 *
Ag 12.5	3.23 ± 0.14 *	4.2 ± 0.1 *	5.6 ± 0.1 *	12.36 ± 0.28 *	6.82 ± 0.04 *
Ag 25	3.26 ± 0.21 *	2.1 ± 0.05 *	4.48 ± 0.4 *	11.52 ± 0.79 *	5.66 ± 1.56 *
Pt 0.625	99.5 ± 1.8	101.2 ± 2.6	108.4 ± 4.5	114.96 ± 2.41	97.30 ± 3.37
Pt 1.25	100.6 ± 2.2	102.7 ± 3.1	107.12 ± 3.4	113.39 ± 4.55	97.7 ± 0.93
Pt 2.5	101.6 ± 4.2	102.4 ± 1.9	103 ± 3.6	109.42 ± 2.4	99.19 ± 0.39
Pt 5	99.2 ± 3.5	105.6 ± 2.1	101.7 ± 5.1	104.61 ± 8.68	103.54 ± 6.43
Pt 10	99.8 ± 1.2	107.2 ± 1.2	102.4 ± 2.1	104.13 ± 1.9	98.18 ± 1.92
Cu 1.56	3.49 ± 0.1 *	67.2 ± 0.4 *	69.7 ± 4.5 *	12.68 ± 0.14 *	101.57 ± 2.32
Cu 3.125	3.36 ± 0.2 *	6.7 ± 0.3 *	5.9 ± 0.3 *	11.94 ± 0.29 *	63.04 ± 6.8 *
Cu 6.25	3.12 ± 0.1 *	5.4 ± 0.1 *	4.7 ± 0.1 *	9.44 ± 0.38	4.43 ± 0.89 *
Cu 12.5	2.42 ± 0.31 *	2.54 ± 0.05 *	3.3 ± 0.05 *	7.75 ± 0.39	3.66 ± 0.2 *
Cu 25	2.34 ± 0.15 *	2.15 ± 0.1 *	2.9 ± 0.1 *	7.09 ± 0.47	2.24 ± 0.22 *
Fe 1.56	101.15 ± 1.9	98.12 ± 4.6	106.6 ± 4.3	113.68 ± 13.62	99.67 ± 1.96
Fe 3.125	101.7 ± 2.2	99.1 ± 5.3	106.4 ± 3.7	104.34 ± 11.2	101.99 ± 2.07
Fe 6.25	103 ± 1.4	96.4 ± 3.2	107.5 ± 1.9	101.97 ± 3.8	102.32 ± 1.23
Fe 12.5	107.6 ± 3.4	94.2 ± 2.7	108.4 ± 4.7	106.24 ± 8.9	100.4 ± 2.25
Fe 25	113.2 ± 2.7	81.2 ± 2.1 *	110.7 ± 7.2	119.28 ± 4.8	101.49 ± 0.92
*p*-value	<0.01	<0.01	<0.01	<0.01	<0.01

* Significant differences in comparison to control (*p* ≤ 0.05); average values ± SD from four repetitions.

**Table 6:** Viability analysis of the microorganisms isolated from ulcerated hoof sores and exposed to AuNP, AgNP, and CuNP complexes.

Nanoparticles (ppm)	*Sphingomonas paucimobilis*	*Ochrobactrum intermedium* I	*Ochrobactrum intermedium* II	*Ochrobactrum gallinifaecis*	*Actinomyces odontolyticus*
**Control group**	100 ± 5.6	100 ± 6.88	100 ± 4.67	100 ± 3.69	100 ± 2.04
AgAu 0.78	11.24 ± 0.25 *	29.7 ± 0.2 *	29.24 ± 0.22 *	36.9 ± 0.13 *	37.89 ± 0.3 *
AgAu 1.56	11.56 ± 0.65 *	9.11 ± 0.58 *	9.59 ± 0.56 *	6.92 ± 0.08 *	8.9 ± 0.12 *
AgAu 3.125	11.75 ± 0.61 *	9.17 ± 0.29 *	9.81 ± 0.7 *	6.84 ± 0.21	5.52 ± 0.76 *
AgAu 6.25	11.23 ± 0.51 *	9.34 ± 0.66	9.01 ± 0.53 *	6.7 ± 0.35 *	3.87 ± 0.58 *
AgAu 12.5	9.68 ± 0.74	11.4 ± 0.38 *	8.15 ± 0.8 *	6.2 ± 0.4 *	2.12 ± 0.05 *
AgCu 0.78	12.5 ± 1.03 *	9.26 ± 0.25 *	15.21 ± 1.1 *	12.54 ± 1.1 *	16.5 ± 0.46 *
AgCu 1.56	8.46 ± 0.51 *	8.53 ± 0.47 *	9.28 ± 1.16 *	6.66 ± 0.96 *	5.88 ± 0.56 *
AgCu 3.125	7.4 ± 0.99 *	7.67 ± 1.35 *	9.85 ± 1.51 *	4.54 ± 0.24 *	4.95 ± 0.95 *
AgCu 6.25	4.9 ± 0.21 *	5.99 ± 0.17 *	5.16 ± 0.21 *	1.92 ± 0.12 *	2.71 ± 0.11 *
AgCu 12.5	3.42 ± 0.71	5.02 ± 0.36 *	4.83 ± 0.36 *	1.1 ± 0.06 *	2.11 ± 0.12 *
CuAu 0.78	12.06 ± 0.78 *	12.18 ± 1.98 *	89.7 ± 6.24	62.13 ± 16.1 *	89.13 ± 0.82
CuAu 1.56	9.45 ± 0.21 *	5.99 ± 0.17 *	9.16 ± 0.21 *	9.16 ± 0.12 *	20.03 ± 2.73 *
CuAu 3.125	9.24 ± 0.9 *	7.5 ± 0.16 *	8.24 ± 0.24	5.69 ± 0.02 *	8.86 ± 0.11 *
CuAu 6.25	2.52 ± 0.71 *	5.02 ± 0.36 *	5.83 ± 0.36 *	3.12 ± 0.01 *	6.71 ± 0.11 *
CuAu 12.5	4.45 ± 0.21 *	4.91 ± 0.3 *	4.56 ± 0.05 *	2.56 ± 0.01 *	3.21 ± 0.05 *
*p*-value	<0.01	<0.01	<0.01	<0.01	<0.01

* Significant differences in comparison to control (*p* ≤ 0.05); average values ± SD from four repetitions.

## Data Availability

Data available on request.
